# Elucidation of the Mechanism of Occasional Anterior Longitudinal Ligament Rupture with Posterior Correction Procedure for Adult Spinal Deformity Using LLIF–Finite Element Analysis of the Impact of the Lordotic Angle of Intervertebral LLIF Cage

**DOI:** 10.3390/medicina59091569

**Published:** 2023-08-29

**Authors:** Hiroki Takeda, Yuichiro Abe, Takaya Imai, Mohd Zaim Mohd Rashid, Daiki Ikeda, Soya Kawabata, Sota Nagai, Kurenai Hachiya, Nobuyuki Fujita, Shinjiro Kaneko

**Affiliations:** 1 Department of Spine and Spinal Cord Surgery, School of Medicine, Fujita Health University, 1-98 Dengakugakubo, Kutsukake-cho, Toyoake 470-1192, Japan; 2 Department of Orthopedic Surgery, Eniwa Hospital, Eniwa 061-1373, Japan; 3 Department of Orthopedic Surgery, School of Medicine, Fujita Health University, Toyoake 470-1192, Japan

**Keywords:** adult spinal deformity, lateral lumbar interbody fusion, anterior longitudinal ligament rupture, finite element analysis, intervertebral cage

## Abstract

*Background and Objectives:* There are several advantages of using lateral lumbar interbody fusion (LLIF) for correction surgeries for adult spinal deformity (ASD); however, we currently have unresolved new issues, including occasional anterior longitudinal ligament (ALL) rupture during the posterior correction procedure. When LLIF was initially introduced, only less lordotic cages were available and ALL rupture was more frequently experienced compared with later periods when more lordotic cages were available. We performed finite element analysis (FEA) regarding the mechanism of ALL rupture during a posterior correction procedure. *Methods:* A spring (which mimics ALL) was introduced at the location of ALL in the FEA and an LLIF cage with two different lordotic angles, 6 and 12 degrees (6DC/12DC), was employed. To assess the extent of burden on the ALL, the extension length of the spring during the correction procedure was measured and the location of the rotation center was examined. *Results:* We observed a significantly higher degree of length extension of the spring during the correction procedure in the FEA model with 6DC compared with that of 12DC. We also observed that the location of the rotation center was shifted posteriorly in the FEA model with 6DC compared with that of 12DC. *Conclusions:* It is considered that the posterior and rostral edge of the less lordotic angle cage became a hinge, and the longer lever arm increased the burden on ALL as the principle of leverage. It is important to use an LLIF cage with a sufficient lordotic angle, that is compatible with the degree of posterior osteotomy in ASD correction.

## 1. Introduction

In recent years, lateral lumbar interbody fusion (LLIF), such as oblique lateral interbody fusion (OLIF) [1] and extreme lateral interbody fusion (XLIF) [2], has been widely used in the surgical treatment of adult spinal deformity (ASD) and lumbar degenerative diseases [1,2,3,4,5,6,7,8,9,10]. There are several advantages of using LLIF for the surgical treatment of these diseases. One of the advantages is relatively solid bone fusion, mostly due to the wider contact area of the LLIF cage with adjacent vertebral bodies, including the cortex area. In the literature, the fusion rate of the anterior body with LLIF has been favorable and reported to be between 93 and 98% [1,2,3,4,5,6,7,8,9,10].

Meanwhile, grade 2 posterior column osteotomy (PCO) is a very effective and widely used procedure to acquire lumbar lordosis for the surgical treatment of ASD [11,12,13]. Additionally, obtaining appropriate lumbar lordosis for any type of lumbar degenerative disease is essential when fusing any segment to achieve long-term goals. This is partly because patients lose their ability to compensate within the area of fusion after surgery. However, one of the potential problems of grade 2 PCO is that the procedure potentially increases the risk of pseudoarthrosis because it removes the facet joint bilaterally. Historically, grade 2 PCO is often combined with posterior lumbar interbody fusion (PLIF) or transforaminal lumbar interbody fusion (TLIF). PLIF/TLIF has been widely used for lumbar interbody fusion; however, these methods have less contact area with adjacent vertebral bodies than LLIF. If LLIF secures anterior bone fusion, we can acquire a relatively large amount of lumbar lordosis by performing multilevel grade 2 PCO without the potential risk of pseudoarthrosis for ASD. We previously showed that LLIF and grade 2 PCO are a good combination to acquire sufficient lumbar lordosis and solid bone fusion for ASD and other lumbar degenerative diseases [13]. We have been applying this combined OLIF grade 2 PCO procedure in the treatment of these diseases and observed a very high percentage of circumferential bone fusion after surgery [13].

**Figure 1:**
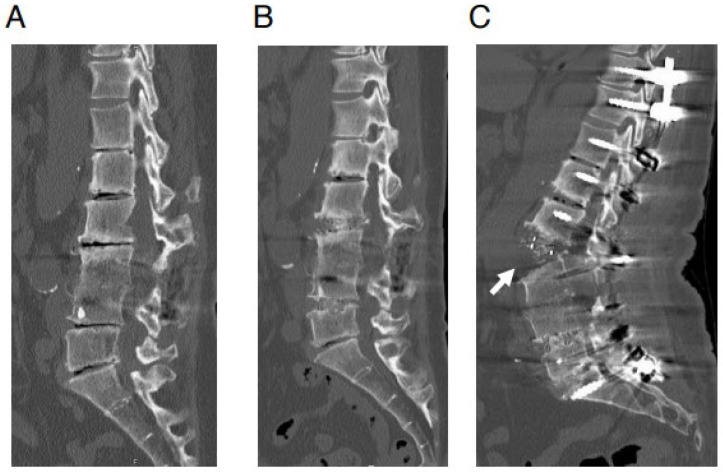
ALL rupture during posterior correction procedure: representative case. A 64-year-old woman with adult spinal deformity. In this case, postero-lateral fusion had been performed at L3/4 level previously at another hospital. Preoperative and postoperative reconstructed sagittal views of CT images. (**A**) Preoperative CT image. (**B**) CT image after OLIF (L2/3·L4/5). (**C**) CT image after posterior correction procedure. In this case, surgery was performed when only the less lordotic (6°) OLIF cages were available. Anterior longitudinal ligament rupture (white arrow) is observed after the posterior correction procedure.

However, we currently have unresolved new issues. One of the new issues is an anterior longitudinal ligament (ALL) rupture which is occasionally observed during the posterior correction procedure for ASD using LLIF (Figure 1) [14,15,16]. The possibility of pseudoarthrosis increases at the level of ALL rupture; therefore, it is very important to prevent it. However, the mechanism of ALL rupture during the posterior correction procedure remains unknown.

In this study, we performed a finite element analysis (FEA) regarding its mechanism. FEA is a method used mainly in industrial fields, such as the construction industry, to perform mechanical simulations using a computer. Based on the design drawings of buildings and automobiles, three-dimensional (3D) models are created on a computer and converted into polyhedral called finite elements, and the stress, deformation, and motion of each element with respect to load and motion are predicted and analyzed [17,18,19,20,21,22,23,24,25,26,27,28,29,30]. When correction surgery for ASD is planned, computed tomography (CT) is usually performed before surgery for diagnosis and treatment, and the surgical plan is also made using 3D measurements constructed from the CT data. By combining the above-mentioned FEA and CT data, it is possible to perform mechanical evaluation and simulation for surgery in various aspects when performing correction surgery for ASD [23,25,29].

The maximum lordotic angle of the OLIF cage is currently 12 or 15 degrees depending on the manufacturing company. However, when the OLIF was initially introduced, the maximum lordotic angle was 6°. When using only the less lordotic 6° OLIF cages, ALL rupture (during the posterior correction procedure) was more frequently observed than in later periods when more lordotic cages were available (Figure 1). Therefore, an LLIF cage with two different lordotic angles (6° and 12°) was employed for comparison in the FEA of the current study. We consider that ALL rupture during a posterior correction procedure is a multifactorial phenomenon. We intended to simplify the experimental method to evaluate the impact of each factor one by one. Additionally, FEA is an effective method for analyzing the cause of ALL injuries during posterior correction procedures in a one-by-one manner. The aim of this study is to evaluate the impact of the lordotic angle of the LLIF cage on the burden to ALL during a posterior correction procedure.

## 2. Materials and Methods

### 2.1. Establishment of the 3D FE Model

Five patients with degenerative lumbar disease were included and analyzed. All patients were women, with an average age of 67.6 years (58–75). For each patient, a custom spinal finite element (FE) model was constructed based on preoperative CT Digital Imaging and Communications in Medicine (DICOM) data. The software ANSYS Workbench 2020 (ANSYS Japan, Tokyo, Japan) was used to model the spine and perform surgical simulations. The raw data collected in the DICOM format were imported into Mimics Research 23.0 (Materialize, Leuven, Belgium) to generate 3D vertebral models in a standard triangle language (STL). Subsequently, the generated STL data were imported into the ANSYS Workbench 2020 in the form of a solid 3D structure, which was built of 10-node tetrahedral element meshes [30]. The model was imported in STL format to 3-matice 15.0, allowing us to inspect the surface model for crisscrosses, tiny channels, or other artifacts (e.g., noise) and repair the triangles in the mesh structure. A smooth function was used to optimize the surface, remove sharp edges, and create a model for the FE mode.

**Figure 2:**
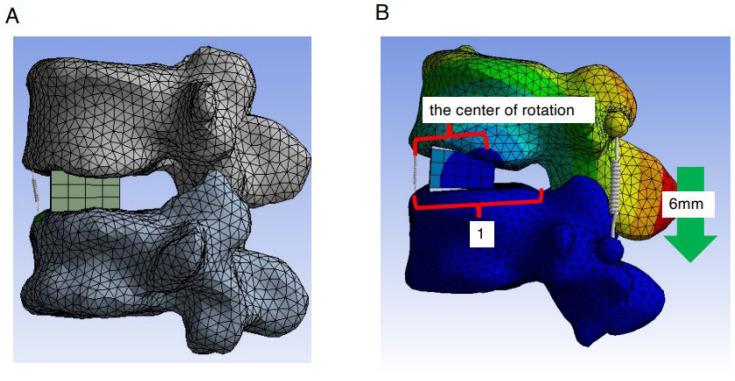
Establishment of FE model at L4/5 level and placement of the OLIF cage and assessment method of the rotation center. (**A**) OLIF cages with a height of 11 mm and a width of 18 mm were added. The L4 caudal endplate and L5 cranial endplate were positioned parallel to each other, and the anterior edges of the OLIF cages were placed 3 mm posterior to the anterior edge of the L5 cranial endplate. To assess the extent of ALL burden during various types of correction procedures, we introduced a spring (which mimics ALL) at the location of the ALL. (**B**) The location of the rotation center was evaluated by calculating the values as follows: the distance between the anterior edge of the L5 cranial endplate and the rotation center was divided by the distance between the anterior and posterior edges.

After importing the established 3D model of the patients into the FE software ANSYS Workbench 23.0, the FE meshing was divided, and OLIF cages with a height of 11 mm and a width of 18 mm were added. The L4 caudal endplate and L5 cranial endplate were positioned parallel to each other, and the anterior edges of the OLIF cages were placed 3 mm posterior to the anterior edge of the L5 cranial endplate. To assess the extent of ALL burden during various types of correction procedures, a spring (which mimics ALL) was introduced at the location of the ALL (Figure 2A). An OLIF cage with two lordotic angles, 6° and 12°, was employed. The elongation length of the spring was measured during the correction procedure to assess the extent of the burden on the ALL. The location of the rotation center was also examined during the correction procedure (Figure 2B). The spring constant was set to 100 N/mm. The coefficient of friction between the end plate and OLIF cage was set to 0.5 referring to a previously reported paper with similar conditions [31] (Figure 2A). This model does not treat cortical bone and cancellous bone separately. It is created as a model that does not consider the deformation for all elements so that the bone fragility in each patient does not affect the result.

### 2.2. Construction of Two Types of FE Models

ALL rupture is a phenomenon occasionally observed in correction surgery for ASD, but not in fusion surgery for degenerative common lumbar diseases. Therefore, two types of FE models were constructed.

**Figure 3:**
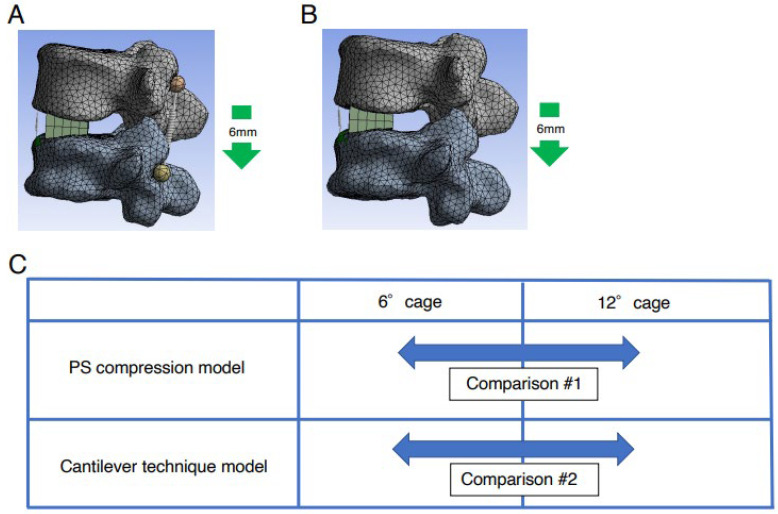
Establishment of PS compression model and cantilever technique model. (**A**) To simulate fusion surgery for degenerative common lumbar diseases, an FE model in which pedicle screws (PSs) were installed was constructed (PS compression model). To simulate the compression procedure in fusion surgery for degenerative common lumbar diseases, the distance between the L4 and L5 screw heads of the PS was reduced by 6 mm. (**B**) A spinous process displacement model was constructed (cantilever technique model) to simulate the cantilever technique in the posterior correction procedure for ASD. With the restraint condition of the L5 vertebral body, a force of 6 mm in the minus *Z*-axis direction was applied to displace the L4 spinous process. (**C**) Comparison between two different degrees of lordotic angle of LLIF cage in two different types of FEA model. An LLIF cage with two different lordotic angles, 6DC and 12DC was used. For both types of LLIF cages, analysis using the PS compression model (comparison #1) and the cantilever technique model (comparison #2) was performed.

In order to simulate fusion surgery for degenerative common lumbar diseases, an FE model in which pedicle screws (PSs) were installed was constructed (PS compression model). With the restraint condition of the L5 vertebral body, a PS with a diameter of 5.5 mm and a length of 45 mm was inserted into L4 and L5. To simplify the model, we assumed that the vertebral bodies and PSs were integrated without considering friction. The Young’s modulus of the vertebral body, OLIF cage, and PSs was set at 12,000 MPa, and Poisson’s ratio was set at 0.3. A spring was used to connect the screw heads at L4 and L5. To simulate the compression procedure in fusion surgery for degenerative common lumbar diseases, the distance between the L4 and L5 screw heads of the PS was reduced by 6 mm, and the elongation degree of the spring at the ALL site and the location of the rotation center were evaluated (Figure 3A). The location of the rotation center was evaluated by calculating the values as follows: the distance between the anterior edge of the L5 cranial endplate and the rotation center was divided by the distance between the anterior and posterior edges (Figure 2B).

A spinous process displacement model was also constructed (cantilever technique model) to simulate the cantilever technique in the posterior correction procedure for ASD. Under the restraint condition of the L5 vertebral body, a force was applied to displace the L4 spinous process by 6 mm in the minus *Z*-axis direction, and the elongation degree of the spring at the ALL site and the location of the rotation center was evaluated (Figure 3B). The elongation degree of the spring at the ALL site and the location of the rotation center were evaluated using the same method as the PS compression model.

### 2.3. Comparison between Two Different Degrees of Lordotic Angle of LLIF Cage in Two Different Types of FEA Model

An LLIF cage with two different lordotic angles, 6 degrees cage (6DC) and 12 degrees cage (12DC), was used. For both types of LLIF cages, analysis using the PS compression models and the cantilever technique model was performed (Figure 3C).

### 2.4. Statistical Analysis, etc.

In the statistical analysis, IBM SPSS Statistics (version 27) (IBM, Armonk, NY, USA) was used, and *p* < 0.05 was considered statistically significant. Student *t*-test was used for all the statistical analysis in this study. The study protocol was approved by the institutional ethics committee.

## 3. Results

### 3.1. Assessment of the Elongation Degree of ALL in the PS Compression Model

**Figure 4:**
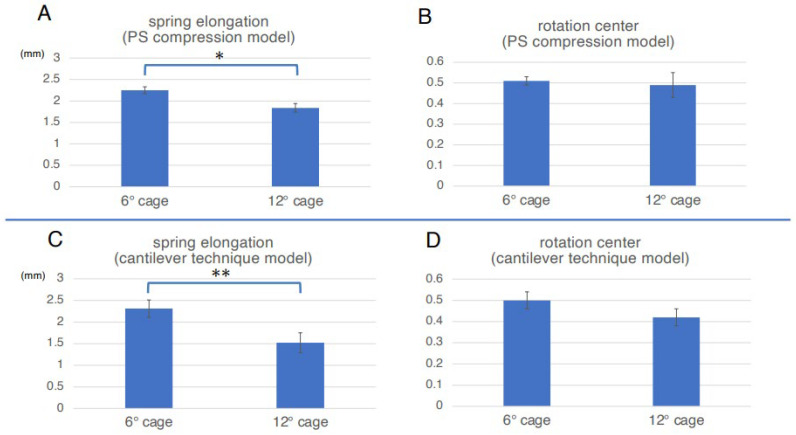
Assessment in two different types of FEA model, the PS compression model (**A**,**B**) and the cantilever technique model (**C**,**D**). (**A**) The spring elongation in the 6DC and 12DC groups was an average of 2.3 mm and 1.8 mm, respectively. The degree of elongation in the 6DC group was significantly larger than that in the 12DC group (* *p* = 0.014). (**B**) The location of the rotation center in the 6DC and 12DC groups was an average of 0.51 and 0.49, respectively. Although the difference between the two groups was not statistically significant, the location of the rotation center in the 6DC group tended to be more posteriorly located than in the 12DC group (*p* = 0.769). (**C**) The average spring elongation in the 6DC and 12DC groups was 2.3 mm and 1.5 mm, respectively. The elongation degree in the 6DC was significantly larger than the 12DC group (** *p* = 0.034). Therefore, in the cantilever technique model (spinous process displacement model), the direct burden to ALL in the 6DC group was considered significantly larger than the 12DC group. (**D**) The location of the rotation center was an average of 0.50 in the 6DC group, and 0.42 in the 12DC. Although the difference between the two groups was not statistically significant, the location of the rotation center in the 6DC group tended to be more posteriorly located than in the 12DC group (*p* = 0.205).

**Figure 5:**
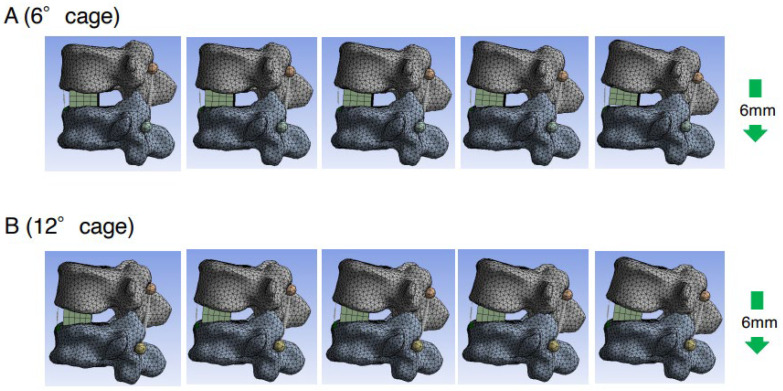
Representative cases with PS compression model. Consecutive images with compression process in PS compression model using 6DC (**A**) and 12DC (**B**) (right side is later phase). It was observed that the posterior edge of the cage became a hinge and the gap between the cage and endplate became obvious in the later phases when a less lordotic cage (6DC) was used.

To assess the direct burden on the ALL, the elongation degree of the spring set at the ALL location was evaluated in the PS compression model. The average spring elongation was 2.3 mm in the 6DC group and 1.8 mm in the 12DC group. The degree of elongation in the 6DC group was significantly larger than that in the 12DC group (*p* = 0.014) (Figure 4A). Representative cases of the PS compression model are shown in Figure 5. It was observed that the posterior edge of the cage became a hinge and the gap between the cage and endplate became obvious in the later phases when using a less lordotic cage (6DC).

### 3.2. Assessment of the Rotation Center Location in the PS Compression Model

To assess the indirect burden on ALL, the location of the rotation center was also evaluated in the PS compression model. The average location of the rotation center in the 6DC group was 0.51, while that in the 12DC group was 0.49. Although the difference between the two groups was not statistically significant, the location of the rotation center in the 6DC group tended to be more posteriorly located than that in the 12DC group (*p* = 0.769) (Figure 4B). Therefore, the indirect burden to ALL in the 6DC group tended to be larger than that in the 12DC group.

### 3.3. Assessment of the Elongation of ALL in Cantilever Technique Model (Spinous Process Displacement Model)

**Figure 6:**
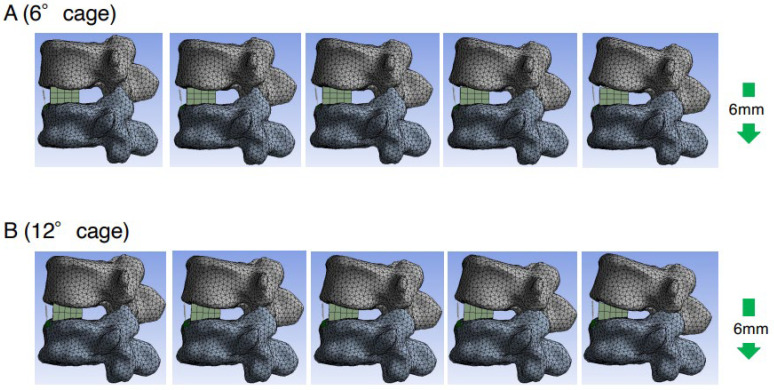
Representative cases with cantilever technique model (spinous process displacement model). Consecutive images with spinous process displacement in the cantilever technique model using 6DC (**A**) and 12DC (**B**) (right side is later phase). It was observed that the posterior edge of the cage became a hinge and the gap between the cage and endplate became obvious in the later phases when a less lordotic cage (6DC) was used.

To assess the direct burden on the ALL, the elongation degree of the spring set at the location of the ALL was evaluated using a cantilever technique model (spinous process displacement model). The average spring elongation in the 6DC and 12DC groups was 2.3 mm and 1.5 mm, respectively. The degree of elongation in the 6DC group was significantly larger than that in the 12DC group (*p* = 0.034) (Figure 4C). Therefore, in the cantilever technique model, the direct burden on the ALL in the 6DC group was considered significantly larger than that in the 12DC group. Representative cases of the cantilever technique model are shown in Figure 6. It was observed that the posterior edge of the cage became a hinge and the gap between the cage, and endplate became obvious in the later phases when using a less lordotic cage (6DC).

### 3.4. Assessment of the Rotation Center Location in Cantilever Technique Model (Spinous Process Displacement Model)

To assess the indirect burden on the ALL, the location of the rotation center was also evaluated using the cantilever technique model. The location of the rotation center in the 6DC group was 0.50 on average, while that in the 12DC group was 0.42. Although the difference between the two groups was not statistically significant, the location of the rotation center in the 6DC group tended to be more posteriorly located than that in the 12DC group (*p* = 0.205) (Figure 4D) Therefore, the indirect burden to ALL in the 6DC group tended to be larger than that in the 12DC group.

## 4. Discussion

LLIF has been widely used in the surgical treatment of ASD in recent years. There are various advantages of applying LLIF to the surgical treatment of ASD. Meanwhile, new issues remain unresolved, including occasional ALL rupture with a posterior correction procedure. The possibility of pseudoarthrosis increases at the level of ALL rupture; therefore, it is very important to prevent it. However, the mechanism of ALL rupture during the posterior correction procedure remains unknown.

When LLIF was introduced initially, only less lordotic cages were available, and ALL rupture with posterior correction procedures was more frequently experienced compared with later periods when more lordotic cages were available (Figure 1). Therefore, we performed FEA regarding the mechanism of ALL rupture with a posterior correction procedure using two different lordotic angle LLIF cages. Furthermore, we developed two different types of FEA models that mimicked the compression procedures for common lumbar degenerative diseases and posterior correction procedures for ASD.

Our results suggest that using an intervertebral cage with sufficient lordosis (12DC) that is compatible with the degree of osteotomy may reduce the burden on the ALL compared with when using a less lordotic cage (6DC).

Maruo et al. reported that ALL injuries occurred in 10 (22%) of 43 patients with ASD during posterior correction procedures who underwent LLIF in advance. They performed a multivariate analysis and reported that XLIF and preexisting osteoporotic vertebral fractures were considered risk factors for ALL injuries [14]. However, ALL injuries during the preceding LLIF procedure were not necessarily noticed. Therefore, it is possible that the ALL had actually been injured and not noticed during the preceding LLIF procedure and was revealed afterward during the posterior correction procedure. In this regard, clinical studies to determine the risk factors for ALL injury during posterior correction procedures have limitations due to the potential contamination of cases in which the ALL was actually injured during the preceding LLIF procedures. Therefore, multivariate analysis using a clinical case series may not be capable of precisely determining the risk factors for ALL injuries during the posterior correction procedure after LLIF procedures for ASD. Additionally, we consider that the cause of ALL injury during the posterior correction procedure is multifactorial.

Taken together, we consider that FEA is an effective method for analyzing the cause of ALL injuries during posterior correction procedures in a one-by-one manner. ALL injury during the posterior correction procedure is sometimes observed in correction surgeries for ASD, but not in fusion surgeries, such as PLIF for common degenerative lumbar diseases. Therefore, in this study, we developed two types of FEA models, the PS compression model and the cantilever technique model which mimics the fusion surgeries for common degenerative lumbar diseases and the correction surgeries for ASD, respectively.

As described in the Results section, we observed that both direct and indirect burdens on ALL in the 6DC group tended to be larger than those in the 12DC group. In addition, the difference in the burden was more obvious in the cantilever technique model than in the PS compression model (Figure 4).

To achieve enough release and to gain appropriate lumbar lordosis for ASD correction, it is important to perform multilevel grade 2 PCO. Grade 2 PCO is also important in the aspect of achieving sufficient central canal and foraminal decompression to prevent secondary canal/foraminal stenosis. It was suggested that when applying a large force, such as spinal deformity correction, the posterior edge of the cage becomes a fulcrum. This phenomenon increases the burden on the ALL when using a cage without a sufficient lordosis angle, which is incompatible with the degree of osteotomy. Taken together, it is important to use an LLIF cage with a sufficient lordotic angle, that is compatible with the degree of posterior osteotomy.

This study had several limitations. First, we performed a single-level analysis to simplify the FEA model as a first step. As a next step, we are planning to perform a multilevel analysis to make the FEA models much closer to clinical cases. Other limitations include the fact that the elastic property of actual ALL is not linear. However, this is a comparative study performing the relative comparison between the two groups with different lordotic angles of the LLIF cage, not a study such as to determine the absolute value of rupture strength, etc. Taken together, we decided to employ the spring to evaluate the burden to ALL to simplify the experimental model. In addition, we consider that the cause of ALL injuries during the posterior correction procedure is multifactorial, as previously mentioned. Therefore, we are planning to assess other possible risk factors regarding the location of the LLIF cage and adjacent fused vertebrae, etc. using FEA as causes of ALL injury during the posterior correction procedure in the future study.

## 5. Conclusions

The present study suggests that when applying a large force, such as spinal deformity correction, the posterior edge of the intervertebral cage becomes a fulcrum and the ALL is subsequently more burdened when a cage without a sufficient lordosis angle, that is not compatible with the degree of osteotomy, is used. Therefore, it is important to use an LLIF cage with a sufficient lordotic angle, that is compatible with the degree of posterior osteotomy in ASD correction.

## Data Availability

The datasets used and/or analyzed in the current study are available from the corresponding author upon reasonable request.

## Abbreviations

LLIF: Lateral lumbar interbody fusion; ASD: Adult spinal deformity; ALL: Anterior longitudinal ligament; FEA: Finite element analysis; DC: Degree cage; OLIF: Oblique lateral interbody fusion; XLIF: Extreme lateral interbody fusion; PCO: Posterior column osteotomy; PLIF: Posterior lumbar interbody fusion; TLIF: Transforaminal lumbar interbody fusion; 3D: Three-dimensional; CT: Computed tomography; FE: Finite element; DICOM: Digital imaging and communications in medicine; STL: Standard triangle language; PS: Pedicle screw.
